# Curcumin Regulates Gut Microbiota and Exerts a Neuroprotective Effect in the MPTP Model of Parkinson's Disease

**DOI:** 10.1155/2022/9110560

**Published:** 2022-11-24

**Authors:** Hong Zhu, Houwen Zhang, Bonan Hou, Bin Xu, Liting Ji, You Wu

**Affiliations:** ^1^Department of Neurology, The Second Affiliated Hospital of Zhejiang Chinese Medical University (Xinhua Hospital of Zhejiang Province), Hangzhou 310000, China; ^2^The Second School of Clinical Medicine, Zhejiang Chinese Medical University, Hangzhou 310000, China; ^3^School of Pharmaceutical Sciences, Zhejiang Chinese Medical University, Hangzhou 310000, China

## Abstract

**Objectives:**

The experiment aimed to explore the effects of curcumin on motor impairment, dopamine neurons, and gut microbiota in the 1-methyl-4-phenyl-1,2,3,6-tetrahydropyridine (MPTP) mice model.

**Methods:**

Mice were randomly assigned to six groups: normal control group, solvent control group, MPTP group, curcumin-low-dose group (40 mg/kg), curcumin-medium-dose group (80 mg/kg), and curcumin-high-dose group (160 mg/kg). After 14 days, each group of mice was subjected to the pole text, the hanging test, and the open-field test. Tyrosine hydroxylase (TH) immunohistochemistry was used to observe the survival of nigrostriatal dopamine neurons. Moreover, ultrastructural changes were observed with a transmission electron microscope in mice striatal tissue cells. Then, 16S rRNA was used to assess changes in the gut microbiota.

**Results:**

(1) Each dose of curcumin reduced pole climbing time and increased suspension score and total distance moved dose-dependently. (2) All curcumin groups improved cell wrinkling and vacuolar degeneration, increased the number of TH positives, improved cell survival, and the higher the dose of curcumin, the better the effect. (3) There were differences in microbiota composition and a relative abundance among the groups. The relative abundance of *Patescibacteria*, *Proteobacteria*, and *Verrucomicrobia* was higher in the MPTP group. The relative abundance of *Patescibacteria*, *Enterobacteriaceae*, *Enterococcaceae* all decreased in all curcumin groups. In addition, the Kyoto Encyclopedia of Genes and Genomes pathways showed a reduction in the superpathway of N-acetylneuraminate degradation after medium- and high-dose curcumin administration.

**Conclusions:**

Curcumin regulates gut microbiota and exerts a neuroprotective effect in the MPTP mice model. This preliminary study demonstrates the therapeutic potential of curcumin for Parkinson's disease, providing clues for microbially targeted therapies for Parkinson's disease.

## 1. Introduction

Parkinson's disease (PD) is one of the most common neurodegenerative diseases with a high incidence and disability rate, which seriously affects the quality of life of patients [1]. Lewy's body composed of *α*-synuclein (*α*-Syn) abnormally gathered in the pars compacta of substantia nigra is an important pathological feature of PD [2]. More and more evidence show that PD is not only a nervous system disease but also a digestive system disease [3].

In the past few decades, human microbiota has become an area of most interest, with many studies focusing on its impact on diseases [4]. Gastrointestinal dysfunction has also been consistently associated with PD and occurs earlier than movement disorders. Gastrointestinal dysfunction can be considered an early biomarker of PD [5]. Moreover, gastrointestinal dysfunction preceding dyskinesia in PD patients may be associated with dysregulation of the “microbe-gut-brain axis.” Lai et al. found changes in the abundance of *Erysipelotrichales*, *Proteobacteria*, *Prevotellaceae*, and *Clostridiales* in intestinal microorganisms by constructing a PD mice model [6]. In PD patients, the level of *Escherichia-Shigella, Proteus, Streptococcus*, and *Enterococcus* were significantly higher than that in healthy individuals [7]. Dysbiosis of the gut microbiota and increased intestinal permeability overstimulate the immune system, leading to a neuroinflammatory response [8]. Then, the *α*-Syn of the enteric nervous system is misfolded and aggregated and passed through the vagus nerve to the dorsal nucleus [9, 10].

One study transplanted gut microbes from PD patients into a healthy mouse model and found that it exacerbated dyskinesia in the mice [3], and antibiotic treatment reversed this effect [11]. In addition, patients with PD constipation who received fecal microbiota transplants saw improvements in their intestinal microbiota imbalance, postural instability, and gait abnormalities [12], suggesting that intestinal microorganisms can be used as targets to improve the condition by changing the composition of intestinal microorganisms.

Curcumin, a polyphenol extracted from the rhizomes of *Curcuma longa*, is converted into biologically active metabolites in the intestine by microbial digestion [13]. Nowadays, it has been shown to have multiple effects such as anti-inflammatory [14], antioxidant [15], anticancer [16], and mitochondrial protection [17]. The bidirectional interplay between curcumin and gut microbes has been demonstrated, as curcumin is not only metabolized by the enzymes of the gut microbes to produce active metabolites but it also strengthens the intestinal barrier and changes the composition of the gut microbes [18].

Curcumin has shown an extremely high therapeutic potential in PD. In animal models, dyskinesia can be improved after the use of curcumin [19]. Many studies have also confirmed that curcumin increased the survival of tyrosine hydroxylase (TH) striatal fibers and nigrostriatal dense part neurons in rat PD model induced by 6-hydroxydopamine (6-OHDA) [20], exhibiting increased dopamine (DA) levels [21]. Concomitantly, curcumin can exert anti-inflammatory effects by inhibiting microglia-mediated neuroinflammation and reducing interleukin-2, chemokines, and cyclooxygenase-2 [22]. Curcumin has also been shown to increase the electrical activity of hippocampal neurons in rotenone-induced PD model rats [23] and regulate the activity of mitochondrial enzyme complex [24]. In addition, a recent clinical study found that curcumin improved motor and nonmotor symptoms and reduced the aggregation of phosphorylated *α*-Syn in skin biopsies in PD patients [25].

However, it is not entirely clear whether curcumin can improve clinical performance by modulating the gut microbes in PD mice. Therefore, in this experiment, 1-methyl-4-phenyl-1,2,3,6-tetrahydropyridine (MPTP) was used to establish PD mice model. We used three different doses of curcumin for the intervention and 16S rRNA gene sequencing to analyze the microbiota diversity in a mice model to see if curcumin could regulate the gut microbes and thus play a therapeutic role.

## 2. Materials and Methods

### 2.1. Drug Preparation

Formulations of MPTP: 100 mg MPTP powder dissolved in 1 mL saline, prepared into 100 mg/mL storage solution, stored in −20°C refrigerator, and diluted to 3 mg/mL before use.

Formulations of low-, medium-, and high-dose solutions of curcumin: 20 mg, 40 mg, and 80 mg of curcumin powder which were accurately weighed added to 50 *μ*l of dimethyl sulfoxide. Then, the mixture was vortexed and shaken until it was completely dissolved. After adding sesame oil for injection to 5 mL, the mixture was stored in a 4°C refrigerator protected from light. The curcumin solution was mixed by vortex shaking before use and prepared into 4 mg/mL, 8 mg/mL, and 16 mg/mL injections, respectively.

### 2.2. Animals

72 clean grade healthy male C57BL/6 mice (6–8 weeks old and 18–22 g) were used in the experiment. Those mice were housed on a 12-hours light/12-hours dark cycle with food and water available ad libitum. Experimental animals, facilities, and feed were provided by the Animal Experiment Center of Zhejiang Chinese Medical University. All experimental protocols were approved by the Experimental Animal Management and Ethics Committee of Zhejiang Chinese Medical University (Ethics Approval No. 20180604-01).

### 2.3. Animal Model Preparation and Grouping

Mice were randomly assigned to six groups: normal control (NC) group, solvent control (SC) group, MPTP group, curcumin-low-dose (CUR-L) group, curcumin-medium-dose (CUR-M) group, and curcumin-high-dose (CUR-H) group. There were 12 rats in each group.

Mice in the MPTP group received a daily intraperitoneal injection of MPTP (30 mg/kg) for 7 consecutive days to establish a subacute PD model. The CUR-L, CUR-M, and CUR-H groups received a combination of both treatments in which curcumin (40 mg/kg, 80 mg/kg, 160 mg/kg) was given 1 hour after administration of MPTP intraperitoneally once a day for 7 consecutive days and every 24 hours after final MPTP for 7 consecutive days. The NC group received a daily intraperitoneal injection of sterile saline (30 mg/kg) for 7 consecutive days. The SC group received a combination of both interventions in which sesame oil containing 1% dimethyl sulfoxide was given 1 hour after administration of sterile saline intraperitoneally daily for 7 consecutive days and every 24 hours after final sterile saline for 7 consecutive days.

### 2.4. Animal Experiments

The following behavioral tests were performed in each group of mice after 14 days. For the pole test, the climbing bar was made with reference to the literature method [26]. The mice were placed at the top of the pole (1 cm diameter and 55 cm height). Then, we corded the time of climbing down the pole. Each animal was tested three times with a minimum of 30 minutes between tests, and the pole climbing time was the average of all three trials. For the hanging test, the mice were strung up by their forepaws on a horizontal wire [27]. The mice were given three points for grasping the wire with both hind paws, two points for one hind paw, one point for not being able to grab the wire with either hind paw, and zero points for dropping directly. The results were calculated as average of three measurements. For the open field test, mice were left in the testing room for 10 minutes to acclimate to the surroundings prior to the trial. The synchronized time recording was started once the mice were lightly placed in the open field box's center. Each mice was measured once for 15 minutes, and the average speed and total distance of movement were recorded. The experiments were conducted from 9 am to 12 pm, and the mice were thoroughly cleaned of feces and urine before the experiments to ensure a clean environment.

### 2.5. Taking Samples and Preparing Tissues

Mice were placed in their respective sterilized cages after the last behavioral test and allowed to defecate freely. Each mice's feces were collected and kept at −80°C in sterile EP tubes after they were generated. Three specimens were taken from each group. Afterward, mice were anesthetized with 0.3% pentobarbital sodium (0.025 mL/g). The right atrial appendage was severed at the same time that a perfusion needle was placed into the left ventricle, and saline (4°C) was quickly infused until the outflow was clear. Four mice in each group were randomly selected to be perfused with 4% paraformaldehyde and then decapitated and the brain was removed. Then, gradient alcohol dehydration and xylene transparent were performed. The tissue blocks were then embedded in wax, serially sliced into 5 *μ*m-thick coronal slices, and stored at 4°C until used for immunohistochemical staining. In each group, two mice were chosen at random, and a striatal tissue that was less than 1 mm ^*∗*^ 1 mm^*∗*^ 1 mm was extracted. For transmission electron microscopy, the tissues were preserved at room temperature after being treated in 2.5% glutaraldehyde.

### 2.6. TH Immunohistochemistry Was Used to Observe the Survival of Nigrostriatal DA Neurons

The prepared tissue sections of each group were subjected to antigen repair by the water bath method, incubated at room temperature, and closed with serum (4°C). Tissue sections were titrated with a mice anti-TH polyclonal antibody (1 : 500) and left overnight (4°C). Hematoxylin was used to stain the sections after diaminobenzidine treatment and secondary antibody incubation was done at room temperature. After that, the sections were fractionated in hydrochloric alcohol, returned to blue, dehydrated in gradient ethanol, and finally sealed. Two fields of view were taken for each mice under a high magnification microscope, and eight fields of view were taken for each group to observe the quantity and morphological alterations in the mice's substantia nigra of TH-positive neurons. The Nano Zoomer digital pathology slide scanner is used to quickly scan slides and convert them into digital sections for data analysis.

### 2.7. The Investigation of the Cellular Ultrastructure Was Conducted Using Transmission Electron Microscopy

The prepared tissue blocks were rinsed 3 times in phosphate buffer, fixed in 1% osmium solution for 1-2 hours, and then rinsed again in phosphate buffer for 3 times and dehydrated in gradient ethanol. The tissue was permeated with an embedding agent gradient, dispensed into 0.5 mL Eppendorf tubes for embedding, and heated at 70°C overnight. Ultrathin sections (50–70 nm) were obtained with a LEICA ultrathin slicer. For observation in transmission electron microscopy, the slices were stained for 15 minutes with lead citrate solution and for 15 minutes with acetate double oxygenic uranium produced in 50% ethanol.

### 2.8. Gut Microbiota Profiling

Using the E.Z.N.A. ®Stool DNA Kit, DNA was extracted from different samples (D4015, Omega, Inc., USA). The whole DNA was eluted in 50 *μ*L of elution buffer and kept at −80°C until quantification in the PCR.

A barcode was added to the 5′ ends of the primers per sample and universal primers were sequenced. PCR amplification was performed in a total volume of 25 *μ*L reaction mixture containing 25 ng of template DNA, 2.5 *μ*L of each primer, 12.5 *μ*L PCR Premix, and PCR-grade water to adjust the volume. The prokaryotic 16S fragments were amplified using PCR under the following conditions: initial denaturation at 98°C for 30 seconds; 32 cycles of denaturation at 98°C for 10 seconds, annealing at 54°C for 30 seconds, and extension at 72°C for 45 seconds; lastly, final extension was performed at 72°C for 10 minutes. With the help of electrophoresis on 2% agarose gels, the PCR products were verified. The PCR products were measured by Qubit and purified using AMPure XT beads. The amplicon pools were ready for sequencing, and the Library Quantification Kit for Illumina and Agilent 2100 Bioanalyzer, respectively, were used to measure the amount and size of the amplicon library. For the library sequencing, the NovaSeq PE250 platform was employed.

### 2.9. Bioinformatics Analysis and Statistical Analysis

On a platform called Illumina NovaSeq, samples were sequenced. Based on each sample's distinctive barcode, paired-end readings were assigned to them and the barcode and primer sequence were removed. Using FLASH, paired-end readings were combined. According to the fqtrim (v0.94), quality filtering on the raw reads was carried out under precise filtering settings to produce the high-quality clean tags. Vsearch software (v2.3.4) was used to screen out chimeric sequences. Using DADA 2, we acquired the feature table and feature sequence after dereplication.

The *R* package (v3.5.2) was used to create the figures. Moreover, QIIME 2 was used to determine the alpha and beta diversity. Sequence alignment was performed using Blast, and each representative sequence's feature sequences were annotated using the SILVA database.

Data were analyzed using SPSS 20.0, and measurement data were expressed as mean ± standard deviation. To evaluate if the data had a normal distribution, the Shapiro–Wilk test was utilized. When comparing more than two groups, one-way ANOVA was used, followed by the LSD test if the variance was homogeneous and the Games-Howell test otherwise.

## 3. Results

### 3.1. Curcumin Improves Motor Dysfunction in PD Mice

**Figure 1:**
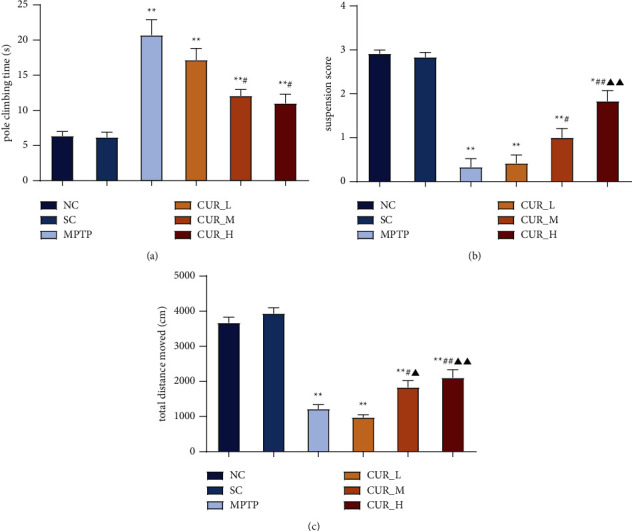
Curcumin improves motor dysfunction in PD mice. (a–c) The motor impairment of mice was tested by the pole text, the hanging test and the open-field test. Data represent the means ± s.e.m; ^*∗*^*P* < 0.05, ^*∗∗*^*P* < 0.01, ^#^*P* < 0.05, ^##^*P* < 0.01, ^▲^*P* < 0.05, ^▲▲^*P* < 0.01. *n* = 12.

To evaluate the ameliorative effect of curcumin on dyskinesia in PD mice, behavioral experiments were performed on mice. The results of the pole text showed that there was no statistical difference between the NC and SC groups. In contrast to the NC group, the pole climbing time was prolonged in the MPTP, CUR-L, CUR-M, and CUR-H groups (*P* < 0.01). Moreover, the differences between the MPTP and CUR-L groups were not significant (*P* > 0.05), but the pole climbing time of mice in both the CUR-M and CUR-H groups was shorter compared to the MPTP group (*P* < 0.05) (Figure 1(a)).

When compared to the NC group, the hanging test showed the elevated scores of the mice in the MPTP (*P* < 0.01), CUR-L (*P* < 0.01), CUR-M (*P* < 0.01), and CUR-H groups (*P* < 0.05). However, no statistically significant difference was seen between the MPTP and CUR-L groups (*P* > 0.05). When compared to the MPTP group, the scores of the mice in the CUR-M (*P* < 0.05) and CUR-H groups (*P* < 0.01) were all improved. Moreover, CUR-H and CUR-L groups differed from one another (*P* < 0.01) (Figure 1(b)).

The open field test showed that the total distance of activity was reduced in both the MPTP group and the CUR-L, CUR-M, and CUR-H groups when compared to the NC group (*P* < 0.01). Similarly, no discernible difference existed between the MPTP and CUR-L groups (*P* > 0.05). In comparison to the MPTP group, mice in the CUR-M and CUR-H groups (*P* < 0.01) had longer total active distances. Additionally, compared to the CUR-L group, the total distance of activity was greater in the CUR-M (*P* < 0.05) and CUR-H (*P* < 0.01) groups (Figure 1(c)).

These results suggested that curcumin improved dyskinesia in PD mice dose-dependently.

### 3.2. Curcumin Reduces MPTP Neurotoxicity and Protects DA Neurons in PD Mice

**Figure 2:**
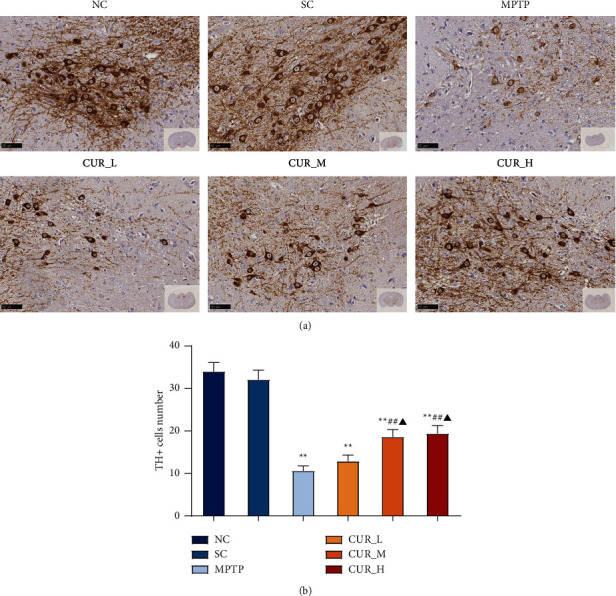
Curcumin rescues the loss of DA neurons in PD mice. (a) Immunohistochemical staining of TH-positive neurons. (b) Quantification of the number of TH-positive cells in the substantia nigra. Data represent the means ± s.e.m; ^*∗∗*^*P* < 0.01, ^##^*P* < 0.01, ^▲^*P* < 0.05, *n* = 8.

To assess the protective effect of curcumin on DA neurons in PD mice, TH immunohistochemically stained positive cells were used to represent the number of DA neurons and could reflect the functional status of DA neurons. The TH-positive neurons in the NC and SC groups were neatly and uniformly arranged, with dark cytoplasmic coloration and intact protrusions. In the MPTP group, TH-positive neurons were sparsely arranged and disorganized, with most of them having only outlines, small cytosolic area, light cytoplasmic staining, and thin and sparse protrusions. TH-positive neurons in the CUR-M and CUR-H groups were relatively dense, with more neatly arranged cells and darker cytoplasmic coloration (Figure 2(a)).

The findings revealed no discernible differences between the NC and SC groups in the quantity of TH-positive neurons in the substantia nigra of the mice. The number of TH-positive neurons in the MPTP, CUR-L, CUR-M, and CUR-H groups were all reduced compared to the NC group (*P* < 0.01). Similarly, the number of TH-positive neurons was higher in the CUR-M and CUR-H group compared to the MPTP group (*P* < 0.01), whereas there was no discernible difference between the MPTP and CUR-L groups (*P* > 0.05). In addition, the number of TH-positive neurons was increased in both the CUR-M and CUR-H groups compared to the CUR-L group (*P* < 0.05) (Figure 2(b)).

**Figure 3:**
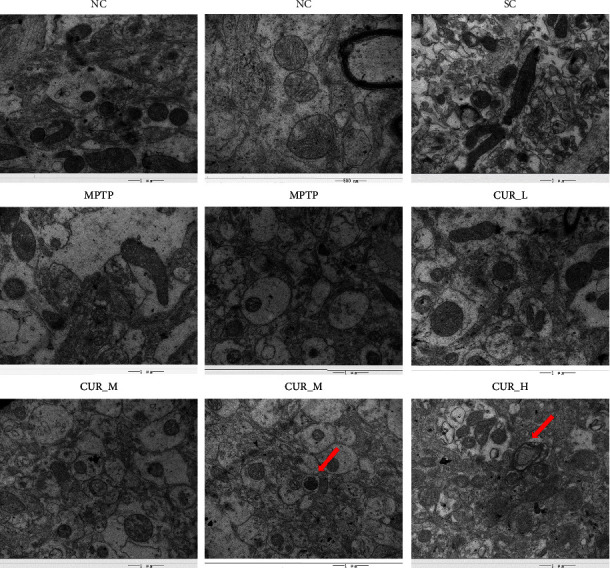
Ultrastructural changes were observed with a transmission electron microscope in mice striatal tissue cells. Curcumin improves cellular wrinkling and vacuolar degeneration. The red arrows indicate the autolysosomes.

Ultrastructural changes were observed with a transmission electron microscope in mice striatal tissue cells. The results showed that the striatal neurons in the NC group were oval in shape, with an intact envelope structure, no intracellular protein aggregation, no obvious cytoplasmic edema or vacuolation, an intact mitochondrial membrane structure, and clearly visible internal cristae. When comparing the SC and NC groups, there was no discernible difference in their electron microscopic performance. The striatal tissue in the MPTP group showed structural destruction of neurons, intracytoplasmic edema, massive vacuolar degeneration, nuclear consolidation, chromatin aggregation, destruction of mitochondrial membrane structure, even swelling and rupture, blurred cristae structure, and no autophagosomes could be observed. The CUR-L group also showed partial structural destruction of neurons, cytoplasmic edema, vacuolar degeneration, blurred mitochondrial cristae, and relatively intact mitochondrial membrane structure compared with the MPTP group. Intracellular edema and vacuolar degeneration were also seen in the CUR-M group, with a relatively intact envelope. When compared with the MPTP group, the mitochondrial cristae in the CUR-M group were clearer, and the mitochondrial membrane results were relatively intact. Autophagic lysosomes are visible in some tissues. Neuronal cell edema and vacuolar degeneration were improved in the CUR-H group compared with the MPTP group. The rest of the CUR-H group behaved similarly to the CUR-M group and autophagic lysosomes were also seen (Figure 3). Curcumin was shown to reduce the neurotoxicity of MPTP in PD mice and protect DA neurons dose-dependently.

The results showed that curcumin improved cell wrinkling and vacuolar degeneration, increased the number of TH positives, and improved cell survival.

### 3.3. Curcumin Regulates the Composition of Gut Microbiota in PD Mice

**Figure 4:**
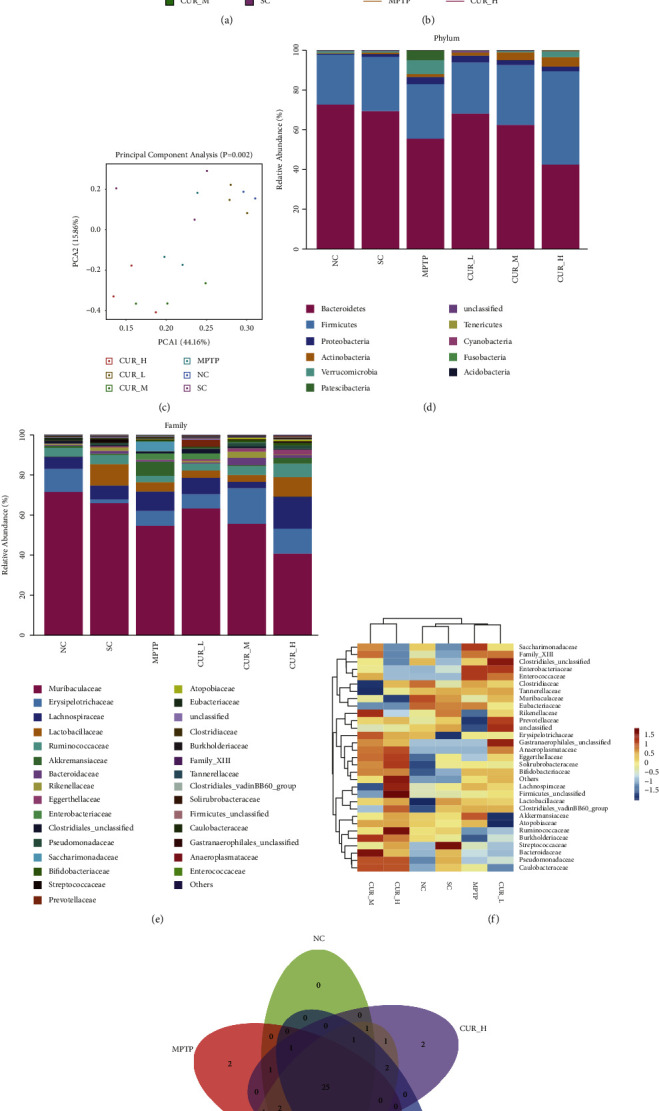
Analysis of 16SrRNA in the gut microbiota of PD mice shows that curcumin regulates the dysregulation of gut microbiota in PD mice. (a) Alpha diversity of gut microbiota based on chao-1. (b) Dilution curves based on the Simpson index. (c) Beta diversity based on PCA analysis. Various colors represent samples of mice from different groups, and the closer the distance between two spots, the smaller the disparity in community composition between the two. (d) Stacked bar graph of relative abundance of gut microbiota at the phylum level. (e) Stacked bar graph of relative abundance of gut microbiota at the family level. (f) A heatmap analysis at the family level. (g) The Venn diagram. Only the top 30 species with the highest relative abundance are shown, and the remaining species are classified as others.

To test our hypothesis that curcumin could improve the gut microbiota of PD mice, we further performed 16S rRNA gene sequencing. Using the QIIME 2 analysis process with better performance, we obtained the Feature data. Alpha diversity was used to assess differences in colony richness and diversity between groups (Figures 4(a) and 4(b)). The results suggest that the samples' microbiota was essentially covered by the sequencing depth and there was no discernible variation between the groups. Beta diversity was further assessed using PCA analysis (Figure 4(c)). The results showed that the SC, NC, and MPTP groups were separated, indicating that there were significant differences in the composition of the microbiota between the SC, NC, and MPTP groups of mice. Notably, the CUR-L, CUR-M, and CUR-H groups were separated from the MPTP group, indicating that curcumin has an effect on the gut microbiota of PD mice.

To further investigate the characteristics of mice gut microbiota, we explored the relative abundance of mice gut microbiota of six groups. At the phylum level, compared with NC group, the relative abundance of *Patescibacteria*, *Proteobacteria*, and *Verrucomicrobia* in MPTP group increased, while the relative abundance of *Bacteroidetes* declined. Compared with the MPTP group, the relative abundance of *Cyanobacteria* in the CUR-L group increased, while the relative abundance of *Patescibacteria* and *Verrucomicrobia* decreased. In the CUR-M group, *Actinobacteria*, *Acidobacteria*, and *Cyanobacteria's* relative abundance increased, but *Verrucomicrobia's* and *Patescibacteria*'*s* relative abundance declined. In the CUR-H group, relative abundance of *Acidobacteria*, *Firmicutes*, *Actinobacteria*, and *Tenericutes* increased while *Bacteroidetes*, *Patescibacteria*, and *Verrucomicrobia* dropped (Figure 4(d)).

At the family level, *Muribaculaceae* dominated, with changes in the relative abundance of various microbiota. Compared with the NC group, the relative abundance of *Enterobacteriaceae*, *Enterococcaceae*, *Saccharimonadaceae*, and *Bifidobacteriaceae* in MPTP group increased, while the relative abundance of *Rikenellaceae*, *Prevotellaceae*, and *Burkholderiaceae* decreased. Compared with the MPTP group, the relative abundance of *Clostridiales-unclassified*, *Rikenellaceae*, *Prevotellaceae*, *Anaeroplasmataceae*, and *Eggerthellaceae* in CUR-L group increased, while the relative abundance of *Saccharimonadaceae*, *Enterococcaceae,* and *Eubacteriaceae* decreased. In the CUR-M group, the relative abundance of *Rikenellaceae*, *Prevotellaceae*, *Erysipelotrichaceae*, *Anaeroplasmataceae*, *Eggerthellaceae*, *Ruminococcaceae*, *Burkholderiaceae*, *Bacteroidaceae,* and *Pseudomonadaceae* increased, while the relative abundance of *Enterobacteriaceae*, *Enterococcaceae*, *Clostridiaceae*, *Tannerellaceae*, *Eubacteriaceae*, *Akkermansiaceae*, and *Atopobiaceae* decreased. In the CUR-H group, the relative abundance of *Prevotellaceaeç Anaeroplasmataceae*, *Eggerthellaceae*, *Solirubrobacteraceae*, *Ruminococcaceae*, *Burkholderiaceae,* and *Pseudomonadaceae* increased, and the relative abundance of *Saccharimonadaceae*, *Family-XIII*, *Clostridiales-unclassified*, *Enterobacteriaceae*, *Enterococcaceae*, *Muribaculaceae,* and *Eubacteriaceae* decreased in relative abundance (Figure 4(e)).

A heatmap analysis was performed based on the relative abundance of each sample at the family level. The abundance of *Enterobacteriaceae* and *Enterococcaceae* was negatively correlated with the dose of curcumin. More importantly, the relative abundance of *Enterobacteriaceae* and *Enterococcaceae* in the CUR-H group has approached that of the NC group (Figure 4(f)).

The Venn diagram suggests that the MPTP, CUR-L, CUR-M, and CUR-H groups all have their own specific gut microbiota (Figure 4(g)). Taken together, these data showed that PD mice have gut microbiota dysbiosis, and curcumin can regulate the distribution and composition of gut microbiota to improve gut microbiota dysbiosis in PD mice.

### 3.4. Analysis of Differences in Gut Microbiota

Utilizing Linear discriminant analysis Effect Size (LEfSe), biomarkers were looked for to identify the distinct bacterial groups within each group. When gut microbiota differences between the groups were analyzed using linear discriminant analysis, the findings revealed a substantial difference in gut microbiota across the groups.

At the phylum level, the relative abundance of *Patescibacteria* and *Verrucomicrobia* in CUR-L is lower than that of the MPTP group, while the relative abundance of *Cyanobacteria* is higher than that of the MPTP group. The CUR-M and MPTP groups did not differ from one another. Moreover, the relative abundance of *Patescibacteria* in CUR-H is lower than that of the MPTP group, while the relative abundance of *Tenericutes* and *Actinobacteria* is higher than that of the MPTP group.

**Figure 5:**
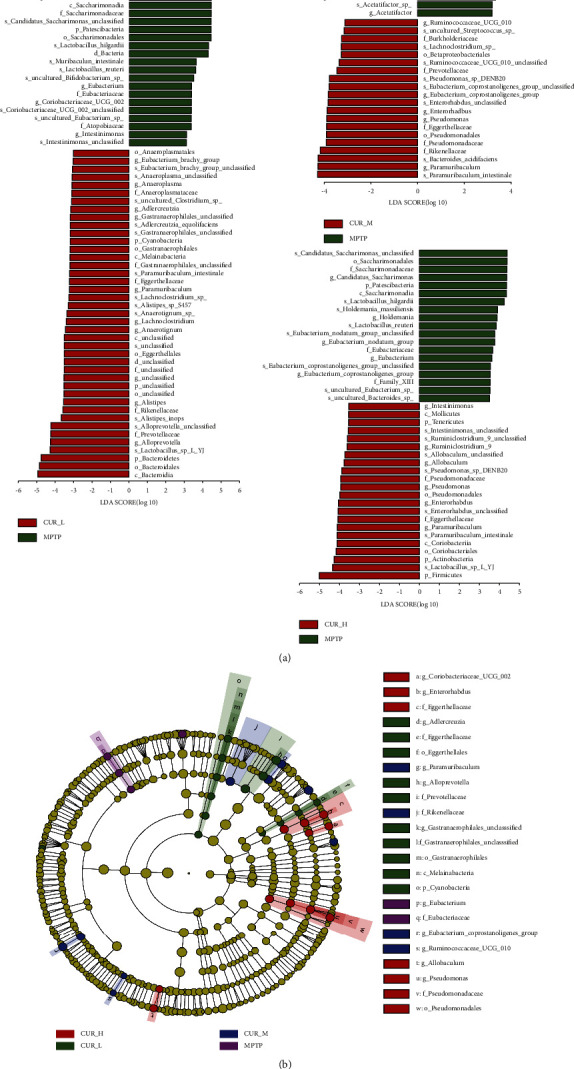
Analysis of species differences by LEfSe. (a) Histogram of LDA value distribution in different treatment groups. The LDA scores (log10) > 3 and *P* < 0.05 are listed; (b) cladogram showing the differential distribution of gut microbiota between the MPTP, CUR-L, CUR-M and CUR-H group. d, domain; p, phylum; c, class; o, order; f, family; g, genus; s, species.

At the family level, there was lower abundance of *Akkermansiaceae*, *Saccharimonadaceae*, *Eubacteriaceaen* and *Atopobiaceae* in the CUR-L group, while *Anaeroplasmataceae*, *Gastranaerophilales-unclassified*, *Eggerthellaceae*, *Rikenellaceae,* and *Prevotellaceae* were more abundant. The relative abundance of *Eubacteriaceae* in CUR-M was lower than that of the MPTP group, while the relative abundance of *Burkholderiaceae*, *Prevotellaceae*, *Eggerthellaceae*, *Pseudomonadaceae*, and *Rikenellaceae* was higher. There was lower abundance of *Saccharimonadaceae*, *Eubacteriaceae*, and *Family-XIII* in the CUR-L group, while *Pseudomonadaceae* and *Eggerthellaceae* were more abundant (Figure 5(a)). In addition, the cladogram showed the differential distribution of gut microbiota between groups (Figure 5(b)).

### 3.5. Prediction of Gene Function of Gut Microbiota

**Figure 6:**
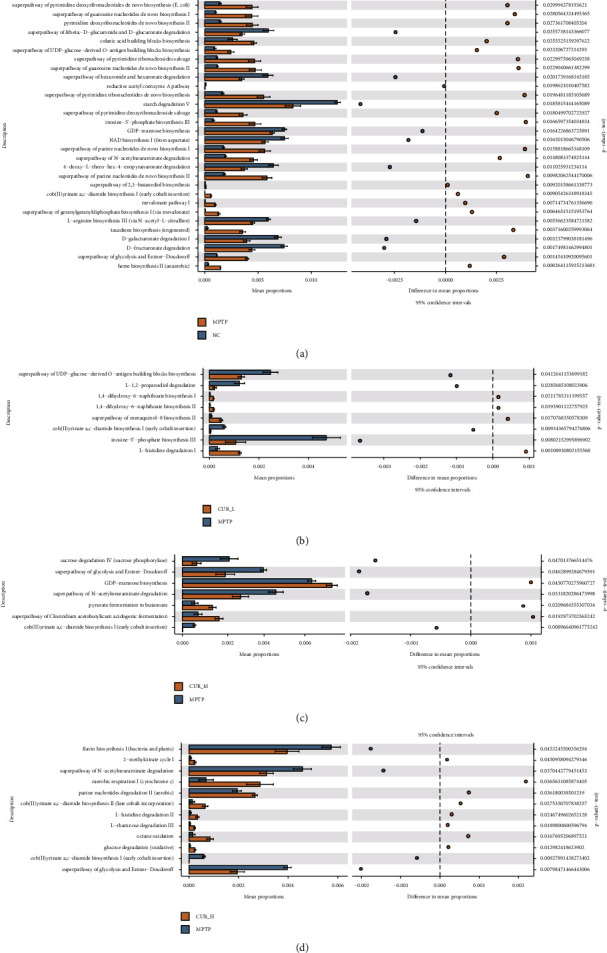
Analysis of KEGG pathway differences between the gut microbiota. (a) KEGG pathway differences between the MPTP and NC group. (b) KEGG pathway differences between the MPTP and CUR-L group. (c) KEGG pathway differences between the MPTP and CUR-M group. (d) KEGG pathway differences between the MPTP and CUR-H group.

The Kyoto Encyclopedia of Genes and Genomes (KEGG) pathways showed substantial group differences by Phylogenetic Investigation of Communities by Reconstruction of Unobserved States 2 (PICRUSt2). There was a significant enrichment of 30 KEGG pathways between the NC and MPTP groups. The administration of curcumin resulted in a number of functional changes in the gut microbiota of mice, such as superpathway of UDP-glucose-derivedO-antigen building blocks biosynthesis, inosine-5′-phosphate biosynthesis III, superpathway of N-acetylneuraminate degradation, cob (II) yrinate a, c-diamide biosynthesis I (early cobalt insertion), superpathway of glycolysis and Entner–Doudoroff and GDP-mannose biosynthesis (Figures 6(a)–6(d)).

## 4. Discussion

As the number of people with PD increases, PD is a growing concern. PD is treated chiefly with drugs such as levodopa preparations, and herbal medicine can play a synergistic role. However, none of the existing therapies can stop the degenerative changes in PD, which forces us to look for new therapies for PD. Curcumin is the main active ingredient of traditional Chinese medicine turmeric, which can be converted into biologically active metabolites in the intestinal tract through microbial digestion after consumption. Thus, curcumin can exert a wide range of pharmacological effects through the “microbe-gut-brain axis” and can be used to treat many chronic diseases [28].

The MPTP-induced PD mice model is more consistent with the course of human PD and partially reflects human PD pathology. Therefore, we explored the therapeutic effects of three different concentrations of curcumin on PD mice by constructing MPTP model mice at 4 mg/mL, 8 mg/mL, and 16 mg/mL. We assessed the motor function of mice by the pole test, hanging test and open field test. It is clear that curcumin improves motor impairment in MPTP mice dose-dependently. In addition, curcumin also reversed the neurotoxicity of MPTP in PD mice. Inhibiting the function of the mitochondrial complex I under the control of monoamine oxidase, MPTP is a lipophilic neurotoxin that easily crosses the blood-brain barrier and causes degenerative necrosis of DA neurons [29]. Coincidentally, curcumin inhibits monoamine oxidase B and protects DA neurons [17]. Our data showed that different doses of curcumin improved DA neuron morphology and number, mitochondrial structure, and neuronal vacuolar degeneration, with higher doses being more effective.

To understand the therapeutic effects of different doses of curcumin on gut microbiota, we used 16S rRNA gene sequencing to analyze the differences in the composition of gut microbiota in each group of mice. From the beta diversity analysis, it is clear that MPTP-induced changes in gut microbiota occurred in the mice model, and different doses of curcumin affected gut microbiota. At the phylum level, the relative abundance of *Patescibacteria*, *Proteobacteria*, and *Verrucomicrobia* was higher in the MPTP group. Following curcumin delivery, *Patescibacteria* and *Tenericutes* showed dose-dependent changes in relative abundance, with *Patescibacteria* decreasing and *Tenericutes* increasing in all groups. The relative abundance of *Cyanobacteria* increased in the CUR-H group.

The changes in *Verrucomicrobia* [30] and *Proteobacteria* [31] abundance in the MPTP group were consistent with the results of previous studies. *Verrucomicrobia* at low concentrations can aid in the intestinal barrier's metamorphosis. However, excessive quantities of *Verrucomicrobia* may increase intestinal leakiness and encourage toxin translocation to the enteric nervous system if the intestinal barrier has been compromised and there is inflammation [30]. Additionally, *Proteobacteria* gives rise to elevated luminal lipopolysaccharide levels in PD, which may impair intestinal integrity [32].

However, there are no reports about the relationship between *Patescibacteria* and PD. *Patescibacteria* is an interesting but currently not widely understood anaerobic bacterium [33]. In the cerebral cortex and hippocampus of a rat model of chronic unpredictable mild stress (CUMS), Zhang et al. found that it was positively associated with 5-hydroxy tryptamine (5-HT), brain-derived neurotrophic factor (BDNF), and glucagon-likepeptide-1 (GLP-1) [34]. Unfortunately, *Patescibacteria* has no relevant studies in animal models of PD. Several studies have confirmed that 5-HT [35], GLP-1 [36], and BDNF [37] all have therapeutic effects on PD model mice. However, in the MPTP group, the relative abundance of *Patescibacteria* in this study increased significantly and decreased with the use of curcumin. It needs to be considered that *Patescibacteria* is an autotrophic microorganism symbiotic with other bacteria [38] and its physiological and biochemical metabolic pathways are not yet clear. Further PD animal model studies are urgently needed to clarify the changes in this microbiota and its neurotransmitters.

*Cyanobacteria* are linked to the etiology of PD because they create *β*-N-methylamino-L-alanine, which depletes glutathione, a key antioxidant, by activating metabotropic glutamate receptors [39]. This neurotoxin is also incorrectly incorporated into human proteins to replace serine, leading to misfolding and aggregation of proteins [40]. The relative abundance of *Cyanobacteria* increased only in the CUR-H group, which may remind us to pay attention to the dose when using curcumin.

At the family level, the relative abundance of *Enterobacteriaceae*, *Enterococcaceae*, *Saccharimonadaceae*, and *Bifidobacteriaceae* was higher in the MPTP group, and the relative abundance of *Rikenellaceae*, *Prevotellaceae*, and *Burkholderiaceae* was lower. After curcumin administration, the relative abundance of *Eubacteriaceae*, *Rikenellaceae*, *Prevotellaceae*, *Anaeroplasmataceae*, *Eggerthellaceae*, *Ruminococcaceae*, and *Burkholderiaceae* all increased and the relative abundance of *Saccharimonadaceae*, *Enterococcaceae*, and *Eubacteriaceae* all decreased. Interestingly, *Enterobacteriaceae* and *Enterococcaceae* has a dose-dependent. It should be noted that the relative abundance of *Enterobacteriaceae* and *Enterococcaceae* in the CUR-H group has approached that of the NC group.

*Bifidobacteriaceae*, which can behave as opportunistic pathogens and induce infection and excessive immunological stimulation in immune-compromised people [41], has been found in higher levels in PD patients in many studies [42]. *Bifidobacteriaceae* can also raise levels of 5-HT [43]; therefore, more research is required to understand its function.

Additionally, the impact of *Enterobacteriaceae* on PD has been the subject of numerous research. *Citrobacter* can induce mitochondrial antigen presentation (MitAP). Recently, *E. coli* has also been shown to cause PD-like motor symptoms in Pink1-/- mice and to produce mitochondria-specificauto-reactive CD8+ T cells via MitAP, causing autoimmunity in DA neurons [44]. Second, *E. coli* can disrupt the distribution of tight junction proteins and actin filaments in intestinal cells, thereby increasing intestinal permeability, disrupting intestinal barrier function [45, 46], and inducing neuroinflammation. In addition, curli, a cell surface amyloid protein abundantly expressed in *E. coli*, promotes *α*-Syn aggregation and increases activation of *α*-Syn overexpressing mouse microglia [47]. In addition, clinical studies have shown that the relative abundance of *Enterobacteriaceae* is positively correlated with the severity of gait difficulties [48]. Combined with our results on motor function and DA neurons, it can be hypothesized that curcumin may protect DA neurons and improve motor impairment in MPTP mice by improving the relative abundance of gut microbiota, such as *Enterobacteriaceae*. More importantly, high doses of curcumin achieve the best results.

We further used KEGG-based functional prediction and found that the function of the mice gut microbiota was quite altered after curcumin administration, such as superpathway of UDP-glucose-derivedO-antigen building blocks biosynthesis, inosine-5′-phosphate biosynthesis III, superpathway of N-acetylneuraminate degradation, cob (II) yrinate a, c-diamide biosynthesis I (early cobalt insertion), and superpathway of glycolysis and Entner–Doudoroff and GDP-mannose biosynthesis. Among them, the superpathway of N-acetylneuraminate degradation is one of the most interesting pathways in this study. Our results showed that the superpathway of N-acetylneuraminate degradation was upregulated in the MPTP group and that this pathway was reduced after curcumin administration.

N-acetylneuraminate is a nine-carbon sugar found in vertebrates and some bacteria. It has been shown that N-acetylneuraminate inhibits amyloid fibrillation and aggregation of *α*-Syn [49], while affecting phosphorylation [50]. Besides. N-acetylneuraminate is components of gangliosides, which can scavenge free radicals and have a significant promotion effect on nerve regeneration. Gangliosides ameliorate the neurotoxicity of MPTP, activate autophagy, and participate in *α*-Syn clearance, thereby reducing the accumulation of *α*-Syn in neuronal cells [51]. Coincidentally, the pathology of PD is characterized by the aberrant accumulation of *α*-Syn in the nigrostriatal dense part of Lewy's microsomes. Our results suggest that curcumin may downregulate N-acetylneuraminate degradation, reduce the accumulation of *α*-Syn, and exert antioxidant effects in the treatment of PD. Notably, this pathway was not significantly different in the CUR-L group, suggesting that there may be a dose-dependent effect of curcumin on this pathway.

There are some limitations of our current study. First, our fecal samples per group of mice were too small and need to be further expanded. Second, because of the low bioavailability of curcumin, it may be able to explore whether its therapeutic effect would continue to increase or remain at a certain level along with further increases in the curcumin dose. Furthermore, curcumin has been shown to inhibit the activation of the PI3K/Akt/mTOR signaling pathway in PD cell models [52]. This foundation allows us to further investigate the mechanism of action of curcumin, which is our area of interest for future research. More in-depth studies are needed to verify our findings.

## 5. Conclusions

Our study shows that curcumin can effectively regulate the changes of gut microbiota, improve the dysfunction of MPTP mice, reduce the neurotoxicity of MPTP in PD mice, and protect DA neurons, and there is some correlation with the dose. This preliminary study demonstrates the therapeutic potential of curcumin for PD, providing clues for microbially targeted therapies for PD.

## Data Availability

The data used to support the findings of this study are included in the article.
